# Ultrasound-Guided Aspiration of a Paralabral Cyst: A Novel Technique for Management

**DOI:** 10.7759/cureus.69072

**Published:** 2024-09-10

**Authors:** Karthik Sriganeshan, Trellane A Willis, William Bonner

**Affiliations:** 1 Department of Translational Medicine, Herbert Wertheim College of Medicine, Florida International University, Miami, USA; 2 Physical Medicine and Rehabilitation, ProForm MD, Miami, USA

**Keywords:** functional impairment, minimally invasive approach, minimally invasive surgical procedures, myasthenia gravis (mg), paralabral cyst, patient-centered care, shoulder joint capsule, suprascapular nerve compression, symptom relief, ultrasound guided

## Abstract

Paralabral cysts of the shoulder joint, though rare, often arise from underlying shoulder pathologies such as labral tears and posterior shoulder capsule instability. These mucin-filled cysts can compress surrounding nerves, particularly the suprascapular nerve, leading to muscle weakness, joint instability, and limited range of motion (ROM). Traditionally, management involves magnetic resonance imaging (MRI) diagnosis followed by surgical repair of the underlying pathology and cyst removal. However, less invasive treatments like ultrasound-guided cyst aspiration have shown promising results. In this case, a 48-year-old male with a history of myasthenia gravis (MG) and chronic bilateral shoulder pain presented with worsening right shoulder pain and weakness during exercise. His extensive treatment history included orthopedic surgery on his left shoulder and multiple Platelet-Rich-Plasma (PRP) injections, which offered only temporary relief. After an MRI confirmed a 2.5 cm paralabral cyst compressing the suprascapular nerve, the patient, opting for a non-surgical approach, underwent ultrasound-guided aspiration. The procedure involved a single aspiration session using a 22-gauge needle under real-time ultrasound guidance, with the complete evacuation of cystic fluid. Follow-up at three and six months revealed complete symptom resolution, with a full recovery of muscle strength and shoulder mobility. No complications were observed, and there was no recurrence of the cyst on follow-up imaging. While surgery remains the gold standard, this case underscores the effectiveness of minimally invasive techniques like ultrasound-guided aspiration, which can offer comparable outcomes with potentially lower recurrence rates and reduced morbidity. Studies support image-guided cyst aspiration as a cost-effective, patient-preferred alternative to surgery, with broader implications for clinical practice in managing similar cases. In summary, paralabral cysts present a complex clinical challenge that benefits from individualized treatment plans. In addition, this case highlights the importance of inter-professional communication and patient-centered care in exploring viable alternatives to surgery, such as ultrasound-guided aspiration, which provides significant symptom relief and functional improvement.

## Introduction

Paralabral cysts of the shoulder joint, mucin-containing structures lined by flat spindle-shaped cells, present an intriguing yet complex challenge concerning treatment options. Surgical removal has traditionally been the predominant first-line treatment for these cysts. However, recent studies have shown that less invasive treatment options can yield results comparable to those of surgical techniques.

The labrum, a ring of fibrous cartilage encompassing the glenoid, the socket of the shoulder joint provides stability and support to the shoulder joint, especially during dynamic or complex movements. The labrum maximizes the stability of the shoulder joint through several mechanisms: deepening the glenoid socket, providing an attachment point for multiple ligaments and tendons, absorbing shock, and enhancing joint congruency. While the etiology of the paralabral cyst has not been fully defined, it has been closely associated with underlying pathologies that disrupt the labrum's stabilizing mechanisms, notably labral tears, posterior shoulder capsule instability, and degenerative changes due to overuse [1]. Disruption of labral stability through these mechanisms allows the synovial fluid to leak from the joint into the tissue surrounding the labrum, forming a paralabral cyst [2]. While typically benign, these cysts can compress the nerves surrounding the labrum, most notably the suprascapular nerve, resulting in joint instability, weakness, and impaired range of motion (ROM) due to supraspinatus and infraspinatus dysfunction [1].

Although ultrasound or computed tomography (CT) scans can provide valuable diagnostic information, magnetic resonance imaging (MRI) is the preferred technique for visualizing the labrum and potential paralabral cysts [3-5]. Historically, surgical removal via an arthroscopic or open surgical approach has been the first-line treatment for a paralabral cyst. Although surgical removal is often successful, these cysts have a high recurrence rate (0%-8% when repaired arthroscopically), which can make surgery a less effective option for some patients and warrants exploration of alternative treatments. Minimally invasive techniques, such as ultrasound-guided aspiration of the cyst, have been shown to be just as effective as surgery and may even provide better outcomes in some cases. A study by Piatt et al. demonstrated that aspiration can be considered on a case-by-case basis, taking into account factors such as cyst size, severity of impingement, and patient demographics. The study found that aspiration provided as much relief and recovery of strength as surgical treatment [1]. Despite these promising findings, there remains a lack of widespread adoption of these techniques, partly due to limited large-scale studies and a prevailing preference for surgical intervention. 

In this case report, we present a patient with a paralabral cyst compressing the suprascapular nerve, successfully treated using ultrasound-guided drainage. This case not only adds to the growing body of evidence supporting minimally invasive techniques but also underscores the importance of precise imaging and individualized treatment strategies in the management of paralabral cysts. By highlighting the successful outcome of ultrasound-guided aspiration in this patient, we aim to contribute to the ongoing discussion regarding the optimal management of this challenging condition and advocate for a broader consideration of minimally invasive options in clinical practice.

## Case presentation

A 48-year-old active male presented to the clinic due to chronic bilateral shoulder pain, with a recent aggravation of the right shoulder associated with pain and weakness in the right shoulder external rotation. His acute right shoulder pain started five days prior when performing overhead movements with dumbbells while exercising and continued over the next five days with no relief. The pain was described as sharp and localized to the posterior aspect of the shoulder, radiating occasionally to the lateral arm. The patient noted significant difficulty in performing daily activities that required shoulder elevation, such as reaching overhead or lifting objects.

The patient’s comprehensive medical history was significant for previous arthroscopic debridement of a paralabral cyst of the left shoulder in 2010, followed by anterior and posterior labral repair of the left shoulder. In 2019, after consultation with an orthopedic surgeon due to similar complaints of right shoulder pain, an MRI revealed mild rotator cuff tendinosis without a tear, mild glenohumeral osteoarthritis, mild biceps tendinosis, a small posterior superior labrum tear, high-grade chondromalacia of the posterior superior glenoid, and a small glenohumeral joint effusion with synovitis within the right shoulder. Despite the recommendation for surgical intervention, the patient opted for a non-surgical approach and received a course of platelet-rich plasma (PRP) injections (three injections given a week apart), which provided substantial relief until the recent exacerbation.

Physical exam

Upon initial evaluation in the clinic, the right shoulder demonstrated a full ROM without pain, but tenderness was noted in the right shoulder's greater tuberosity. Notably, the patient exhibited significant weakness and instability during resisted external rotation, both in adduction and at 90 degrees of abduction, with strength graded as 2/5. The Hawkins and Neer tests, used to assess for subacromial nerve impingement, were mildly positive. These findings, coupled with the patient’s history and recent aggravation, led to an initial differential diagnosis that included a recurrent paralabral cyst, rotator cuff pathology, or subacromial impingement syndrome. The patient was prescribed naproxen for pain management and referred for an MRI to confirm the diagnosis.

Imaging

**Figure 1:**
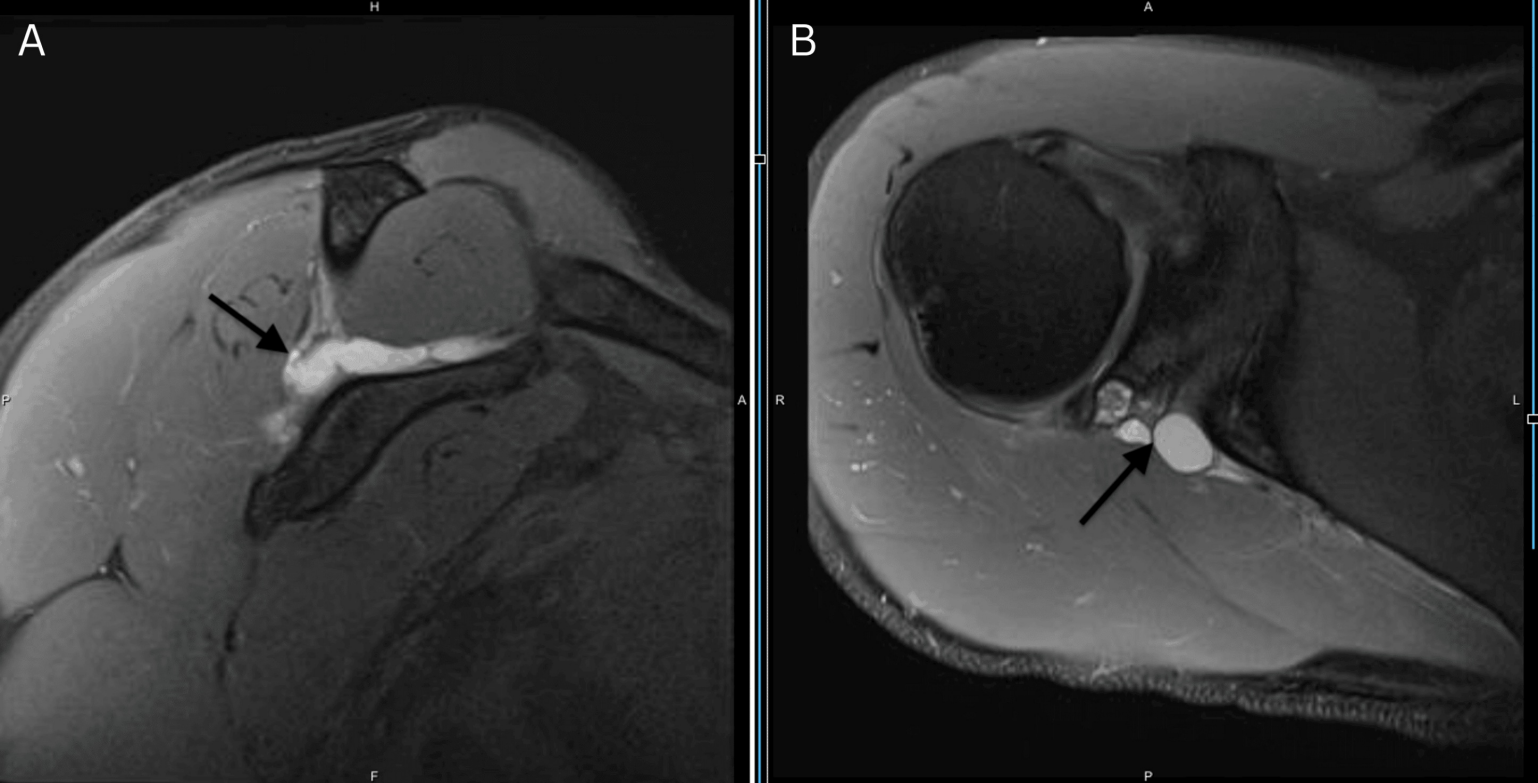
Pre-aspiration MRI image: MRI image showing a paralabral cyst compressing the suprascapular nerve (A) Pre-aspiration coronal MRI image showing a paralabral cyst compressing the suprascapular nerve. An arrow pointing at fluid collection causes nerve impingement. (B) A pre-aspiration axial MRI image shows a paralabral cyst compressing the suprascapular nerve.

MRI revealed a multi-septated cystic lesion seen adjacent to the posterior labrum, which extends from the 3- to 5-o’clock position, consistent with a paralabral cyst measuring 2.9 cm in length (Figure 1). Additionally, a tear of the posterior labrum was identified in the same region. The patient also had mild arthritic changes in the glenohumeral joint, mild supraspinatus tendinopathy without tear, and degenerative changes in the acromioclavicular joint. Considering the patient’s prior surgical history and preference to avoid another surgical intervention, a discussion of treatment options ensued. These included repeat PRP injections, physical therapy, or ultrasound-guided aspiration of the cyst. Given his previous success with non-surgical management but recent lack of sustained relief, the patient elected to proceed with ultrasound-guided aspiration. 

Procedure

The details of the procedure, alternative options, risks, and benefits were discussed with the patient, and appropriate consent was obtained. The procedure site was identified and marked under sterile conditions. A linear ultrasound probe was used to locate the paralabral cyst, situated just medial to the glenohumeral joint and inferior to the spine of the scapula. After local anesthesia was administered using a 30-gauge needle, a 16-gauge needle was inserted in-plane to aspirate 3.5 mL of serosanguinous synovial fluid from the cyst. The patient reported immediate pain relief during shoulder flexion and abduction post-procedure. A sterile dressing was applied, and the patient was given post-procedure care instructions, including pain management and a physical therapy regimen. A follow-up appointment was scheduled for two weeks later.

Follow-up

**Figure 2:**
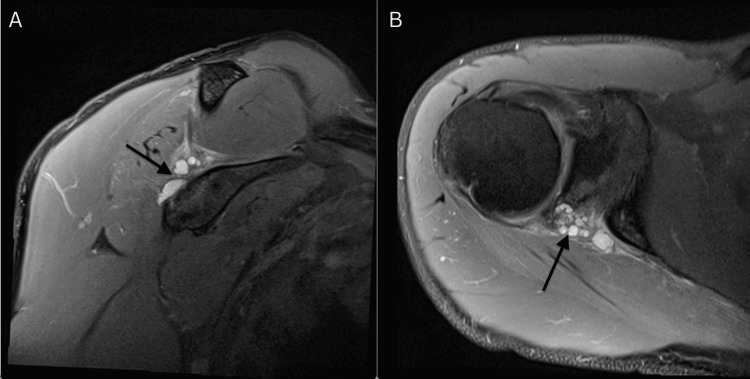
Post-aspiration MRI image: post-aspiration MRI demonstrating the resolution of the paralabral cyst and alleviation of nerve compression (A) Post-aspiration coronal MRI image demonstrating the resolution of the paralabral cyst and alleviation of nerve compression. (B) Post-aspiration axial MRI image demonstrating the resolution of the paralabral cyst and alleviation of nerve compression. Arrow shows a significant decrease in fluid collection.

After two weeks, the patient returned, reporting significant improvement in his pain and ROM, with his strength improving to a 3/5. However, he continued to experience some discomfort in the posterior shoulder, prompting further evaluation with ultrasound and electromyogram (EMG). The ultrasound revealed a small re-accumulation of fluid around the suprascapular nerve, significantly less than the initial collection (Figure 2). EMG confirmed significant denervation of the infraspinatus muscle, consistent with suprascapular nerve impingement at the spinoglenoid notch.

Patient’s perspective and current condition

The patient expressed satisfaction with the significant reduction in pain and improvement in function but remained concerned about the residual discomfort and weakness. He was particularly interested in non-surgical options, having had positive experiences with minimally invasive treatments in the past.

Assessment and next steps

Given the patient’s complicated medical history, significant improvement in pain, improvement in muscle strength, and persistence in non-surgical management, the decision was made to repeat a second ultrasound-guided aspiration of the residual fluid. The same technique as mentioned prior was performed, and 2 mL of serosanguinous fluid was aspirated. In the following weeks, the patient reported complete pain resolution and improvement of both ROM and muscle strength with physical therapy. Further testing elicited improvement in infraspinatus strength from 3/5 to 4+/5 following the second aspiration along with an almost complete range of motion.

## Discussion

A paralabral cyst is a fluid-filled sac that develops near the labrum of the shoulder joint. Observed in 2%-4% of the general population, these cysts are particularly prevalent in men in their third and fourth decades of life and are most commonly due to underlying shoulder joint pathologies, including labral tears, posterior shoulder capsule instability, and degenerative changes due to overuse of the joint [6]. The labrum, a crucial structure consisting of fibrous cartilage encircling the glenoid (shoulder joint socket), plays a vital role in stabilizing and supporting the shoulder joint during dynamic and complex movements. Its functions include deepening the glenoid socket, providing attachment points for various ligaments and tendons, absorbing shock, and enhancing joint congruency. Disruptions in labral stability, often due to injuries or overuse, allow synovial fluid from the joint to accumulate in the surrounding tissue, forming a paralabral cyst [2]. While paralabral cysts are generally considered benign, their impact can vary depending on their size and location. Especially when these cysts compress nerves around the labrum, particularly the suprascapular nerve, they can lead to joint instability, weakness, and impaired ROM, often manifesting as dysfunction in the supraspinatus and infraspinatus muscles [1].

The suprascapular nerve, derived from the upper trunk of the brachial plexus (formed by C5 and C6 nerve roots), provides motor innervation to the supraspinatus and infraspinatus muscles. This innervation is essential for shoulder movements such as abduction and external rotation [7]. The nerve travels through the supraspinatus fossa, where it becomes susceptible to damage and impingement at two locations: the superior passage through the scapular notch and the posterior passage at the spinoglenoid notch. Impingement of the suprascapular nerve at these locations can result in specific patterns of muscle weakness. Superior impingement often correlates with both supraspinatus and infraspinatus weakness, while inferior impingement is typically associated solely with infraspinatus weakness [8]. Paralabral cysts are identified as one of the common causes of impingement along the inferior pathway.

Our case demonstrates pathology involving compression of the suprascapular nerve at its posterior passage through the spinoglenoid notch, resulting in infraspinatus dysfunction. The patient exhibited weakness in external rotation at both adducted and abducted positions, raising suspicion of suprascapular nerve impingement along the infraspinatus innervation pathway. The involvement of the supraspinatus was ruled out through the empty-can test during the physical examination. While physical examination maneuvers can aid in diagnosing shoulder joint pathology, imaging studies are essential for definitive diagnosis. Electromyography and nerve conduction studies are the gold standard for diagnosing suprascapular nerve pathology. Subsequent imaging studies via CT scan and MRI confirm impingement caused by soft or bony tissue damage [7].

Treatment options for these cysts vary greatly depending on the extent of patient pain and the degree of function loss. In less severe cases, symptomatic pain management via medication (e.g., NSAIDs) and rehabilitation through physical therapy can alleviate pain and preserve functionality. However, with more complex and severe cases, conservative treatment may not be viable. Given that paralabral cysts often occur in conjunction with an inciting injury, like the labral tear in this case, the gold standard treatment is arthroscopic or open surgery. The procedure typically involves suture repair of the labrum and decompression or removal of the cyst. Despite the success of surgery, recurrence rates, and patient hesitancy, as observed in this case, present challenges, prompting exploration of alternative approaches.

Ultrasound-guided aspiration has emerged as a viable non-surgical alternative, offering advantages such as reduced invasiveness, lower complication rates, and potentially comparable outcomes to surgery. In our case, subsequent follow-up revealed a recurrence of fluid around the suprascapular nerve, a common challenge in managing paralabral cysts [9,10]. The decision to repeat ultrasound-guided aspiration was grounded in the patient's positive response to the initial procedure and his commitment to non-surgical management. This repeated approach proved successful, leading to complete symptom resolution and improvement in both ROM and muscle strength. Follow-up examination after the second aspiration showed no fluid re-accumulation and full symptomatic relief. 

Despite the success of ultrasound-guided aspiration in this case, it is important to consider the limitations and potential long-term outcomes associated with this technique. Recurrence of cystic fluid is a well-documented complication, as demonstrated in our patient’s case. The mechanisms of recurrence may involve incomplete aspiration of the cyst, ongoing synovial fluid leakage due to persistent labral pathology, or underlying joint instability. While repeat aspirations, as performed in this case, can be effective in managing recurrences, they may not be suitable for all patients, particularly those with large or complex cysts. Additionally, the long-term outcomes of ultrasound-guided aspiration remain less well-characterized compared to surgical intervention. Although studies such as Piatt et al. have shown promising short-term results, more extensive research is needed to establish the durability of this approach over time and its impact on recurrence rates [11].

In terms of patient-centered considerations, the decision to pursue ultrasound-guided aspiration was strongly influenced by the patient’s previous experiences with surgery and his preference for less invasive treatment options. The success of the aspiration in providing immediate pain relief and functional improvement was aligned with the patient’s goals and highlights the importance of individualized treatment plans that consider patient preferences and medical history. However, it is also essential to educate patients about the potential for recurrence and the possibility that further interventions, including surgery, may be necessary if non-surgical approaches fail to provide sustained relief.

It is also critical to consider the patient’s history of myasthenia gravis (MG), an autoimmune neuromuscular disorder that could have contributed to the weakness in his shoulder muscles. MG is characterized by fluctuating muscle weakness, particularly affecting muscles that are used repetitively or for sustained periods. In this patient, the pre-existing weakness due to MG could have exacerbated the effects of the suprascapular nerve compression, making the muscle dysfunction more pronounced. Additionally, MG may have played a role in the initial injury by predisposing the patient to muscle fatigue and overuse, which could have led to the labral tear and subsequent formation of the paralabral cyst. The presence of MG also complicates the interpretation of diagnostic studies, as muscle weakness attributed to nerve impingement might be difficult to distinguish from the baseline weakness associated with MG. This underscores the importance of a comprehensive approach to diagnosis and treatment, taking into account the potential interactions between MG and other musculoskeletal conditions.

By aspirating the fluid of the paralabral cyst, we effectively eliminated the source of impingement. With the appropriate physical therapy regimen and proper management, our patient fully relieved his pain and regained full function in his right shoulder. Using ultrasound for both diagnosis and as a real-time guide during aspiration allows for targeted, accurate removal of cystic fluid, minimizing complications and optimizing outcomes. The success of this procedure in our case, as evidenced by the immediate relief of symptoms, aligns with findings from studies like that of Piatt et al., which advocate for considering aspiration based on factors such as cyst size, impingement severity, and patient demographics. Other studies have demonstrated that image-guided aspiration of paralabral cysts is not only cost-effective but also highly effective when compared to surgical intervention [12]. In a retrospective study of 15 patients who underwent ultrasound-guided cyst aspiration, 86% reported symptom resolution over a follow-up period of two to four months [5]. Similarly, a study by Hashimoto et al. involving four patients with paralabral cysts and impingement symptoms reported symptom resolution after ultrasound-guided aspiration [12].

However, given the potential for recurrence and the limited data on long-term outcomes, further research is warranted to better understand the role of ultrasound-guided aspiration in the management of paralabral cysts. Clinicians should weigh the benefits of less invasive techniques against the possibility of recurrence and tailor their approach to the individual patient, taking into consideration factors such as medical history, patient preferences, and the specific characteristics of the cyst.

## Conclusions

This case report contributes to the evolving understanding of paralabral cyst management by highlighting the successful use of ultrasound-guided aspiration as a viable and effective alternative to surgical intervention. The patient experienced significant symptom relief, improved range of motion, and enhanced muscle strength following the procedure, even in the context of a complicated medical history, including MG and prior surgical interventions. These results suggest that ultrasound-guided aspiration should be considered, particularly, for patients who prefer non-surgical approaches or who present with recurrent symptoms after previous treatments. From a broader clinical perspective, this case underscores the importance of individualized treatment strategies, where patient history, preferences, and the specific characteristics of the pathology are carefully weighed. The success of ultrasound-guided aspiration in this case aligns with emerging evidence that supports its use as a first-line or adjunctive treatment, especially in cases where surgical risks outweigh potential benefits. This approach not only reduces the invasiveness of treatment but also minimizes recovery time and associated complications, making it an attractive option for a subset of patients.

To further refine treatment algorithms and enhance patient outcomes, several specific areas for future research should be pursued. Long-term studies are needed to compare the recurrence rates and overall efficacy of ultrasound-guided aspiration versus surgical interventions. Additionally, research should focus on identifying patient-specific factors, such as cyst size, location, and underlying joint pathology, that may predict the success or failure of aspiration techniques. Investigating the role of ultrasound-guided aspiration in patients with neuromuscular disorders, such as MG, could also provide valuable insights into tailoring treatments for this population.

Clinicians are encouraged to integrate these findings into practice by considering ultrasound-guided aspiration as a primary treatment option in appropriate cases. Clear inter-professional communication, particularly among orthopedic surgeons, physical medicine and rehabilitation specialists, and physical therapists, is crucial to ensure that patients receive comprehensive, coordinated care. By staying informed about the latest evidence and continuously evaluating patient outcomes, clinicians can make more informed decisions and offer personalized, effective treatment plans.
